# Neural leprosy investigation using electroneuromyography and the ML Flow rapid test: a case report

**DOI:** 10.1590/0037-8682-0586-2023

**Published:** 2024-02-12

**Authors:** Roque Rafael de Oliveira, Gabriela Belmonte Dorilêo, Ana Carolina da Silva Ferreira Mendonça, Amílcar Sabino Damazo

**Affiliations:** 1 Universidade Federal de Mato Grosso, Faculdade de Medicina, Programa de Pós-Graduação em Ciências da Saúde, Cuiabá, MT, Brasil.; 2 Clínica Idea, Instituto de Dermatologia, Cuiabá, MT, Brasil.; 3 Secretaria Municipal de Saúde de Nova Mutum, Nova Mutum, MT, Brasil.; 4 Clínica de Neurofisiologia e Reabilitação Ltda, Cuiabá, MT, Brasil.; 5 Universidade Federal de Mato Grosso, Faculdade de Medicina, Departamento de Ciências Básicas em Saúde, Cuiabá, MT, Brasil.

**Keywords:** Bacilloscopy, Electroneuromyography, Serology

## Abstract

Neural leprosy, which is characterized by nerve involvement without visible skin lesions, presents a diagnostic challenge. This case report examined the significance of diverse diagnostic modalities in the identification of pure neural leprosy. A 28-year-old patient with symptoms of edema, pain, paresthesia, and diminished sensitivity in the lower limbs underwent various tests. A stilt skin smear yielded negative results on bacilloscopy, whereas a Fast ML Flow leprosy test and electroneuromyography supported the diagnosis. This discussion highlights the importance of accessible methods for early investigation. This study emphasizes the multidisciplinary approach and value of the Fast ML Flow leprosy test and electroneuromyography for diagnosing neural leprosy.

## INTRODUCTION

Neural leprosy is a variant of the disease characterized by nerve involvement in the absence of visible cutaneous lesions^1^. Given its neuropathic nature, the initial clinical signs may manifest subtly, such as muscular contractions, paresthesia, and localized areas of hypoesthesia^2^. These indications often go unnoticed by patients. 

A thorough search of the Cochrane Library, LILACS, SciELO, MEDLINE, PubMed, and PubMed Central databases was conducted to identify relevant studies on neural leprosy, including clinical presentations, diagnostic approaches, and therapeutic interventions. Conventional bacilloscopic examination of slit-skin smears exhibits diminished sensitivity in such cases. Molecular diagnostics offer enhanced accuracy, but are financially burdensome and predominantly accessible solely at specialized medical facilities^3^. Some studies have shown that qPCR tests in slit-skin smears are positive in almost 80% of cases^4^.

Recently, rapid leprosy testing has emerged as a highly efficacious and minimally invasive alternative that facilitates the assessment of contacts in confirmed cases. Recently, the use of serological kits as diagnostic tools for neural leprosy has emerged as a promising approach. The anti-PGL1 IgM serology ELISA was a great advancement, being positive in 52% of multibacillary cases^4^ ^,^ ^5^. The use of the ML Flow test as a criterion for classification in one study increased to 11% of patients requiring treatment for multibacillary leprosy in Brazil^6^ ^,^ ^7^.

In neural leprosy, a detailed clinical examination is essential. However, neural thickening is not always recognized, confirming the need for a combined assessment^8^. Abnormalities observed on electroneuromyography may be present in a high proportion of asymptomatic patients with leprosy. Several studies have shown that sensory nerve conduction impairment is the most frequent and earliest detected parameter in electroneuromyography evaluation^9^ ^-^ ^11^. 

This case report highlights the importance of employing diverse diagnostic modalities as invaluable tools for the identification and management of neural leprosy.

## CASE REPORT

A 28-year-old individual from Nova Mutum/MT/Brazil (January/2022), presented with edema, pain, paresthesia, and diminished thermal and tactile sensitivities in the right lower limb. When asked about familial history of leprosy, the patient cohabited with a sibling who had undergone leprosy treatment a decade ago. During physical examination, edema was observed in the right foot, along with thickening and tenderness upon palpation of the right common fibular and posterior tibial nerves. Additionally, the patient exhibited no tactile sensitivity on the lateral side of the leg and the dorsal and plantar regions of the right foot, which are innervated by the fibular and posterior tibial nerves, when evaluated using an orange esthesiometer. 

**FIGURE 1:**
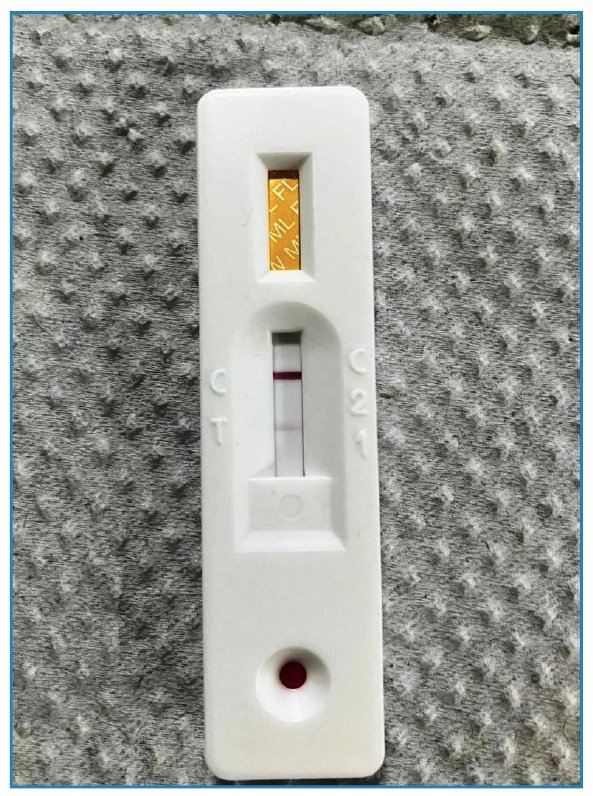
Positive result of ML Flow serological test for leprosy.

Bacilloscopy slit-skin smear microscopy was conducted on both auricular lobes and elbows, yielding negative results. However, a rapid leprosy test (Bioclin fast ML flow leprosy, Brazil) was performed, which provided a positive result (Figure 1), further supporting the suspected diagnosis. 

Mild motor dysfunction was also evident in the foot, as evidenced by the difficulties encountered while performing resistance maneuvers.

**FIGURE 2:**
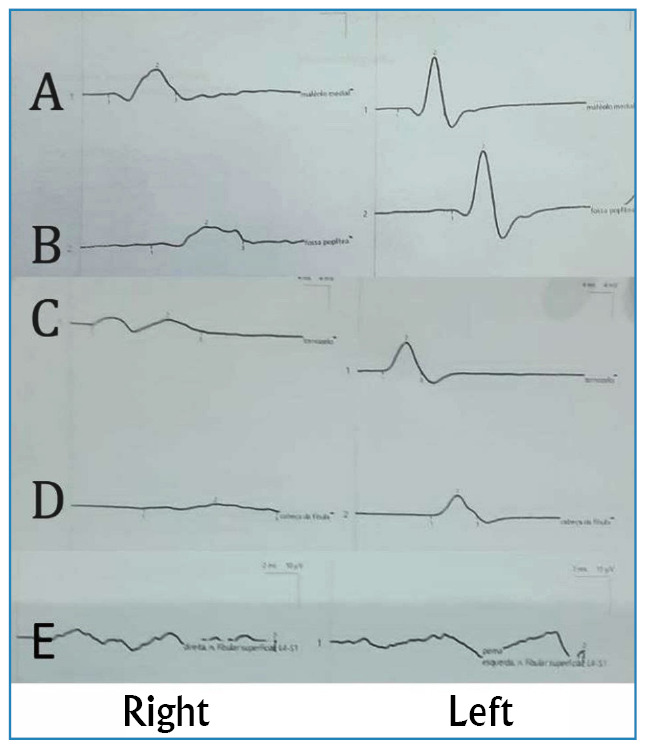
Electroneuromyographic evaluation of neural leprosy patient. Action potentials have normal and symmetrical amplitude, latency and velocity in the posterior tibial and common fibular nerves on the left. Action potentials present with dispersion in the posterior tibial and common fibular nerves on the right. Absence of sensory potentials. **(A)** Medial malleolus. **(B)** Popliteal fossa. **(C)** Ankle. **(D)** Head of fibula. **(E)** Fibular superficial nerve.

Electroneuromyography was performed to further evaluate the nerves, revealing a pattern consistent with multiple motor and sensory mononeuropathy, characterized by the dispersion of motor potential and absence of sensory potential (Figure 2). This was characterized by motor potential dispersion and absence of sensory potential in the right common fibular and tibial nerves. 

Based on the clinical presentation, sensitivity tests, electroneuromyography results, and positive rapid leprosy test outcomes, the patient was diagnosed with pure neural leprosy. Treatment with the recommended multidrug therapy for leprosy was promptly initiated (PQTU).

## DISCUSSION

The availability and accessibility of diagnostic methods for leprosy still pose challenges in reaching the general population because of the requirement for specialized equipment and trained personnel. However, advancements such as fast ML Flow leprosy have expanded the prospects of diagnosing patients efficiently^5^ ^-^ ^7^. This report focuses on a case of pure neural leprosy, a clinical variant presenting with a complex diagnostic scenario, characterized by neurological impairment without apparent skin lesions^1^. 

Although bacilloscopy is a crucial complementary examination for diagnosing leprosy, it yields negative results. Bacilloscopy sensitivity is reduced in cases of neural leprosy, which may result in false-negative outcomes^3^.

Additionally, the Fast ML Flow leprosy test yielded a positive result in this case. This is an IgM antibody detection test against the PGL-1 antigen (phenol glycolipid-1), which plays a pivotal role in facilitating early diagnosis, with a sensitivity of 90% and specificity of 98%^5^ ^-^ ^7^. It is a minimally invasive procedure that expedites initiation of treatment^3^.

These findings strongly suggested a condition compatible with neural leprosy. Electroneuromyographic evaluation revealed signs of multiple motor and sensory mononeuropathies. This test has gained prominence as an early diagnostic tool for leprosy^8^ ^-^ ^11^.

In conclusion, this case emphasizes the significance of a multidisciplinary approach, which is essential for diagnosing neural leprosy.
